# HRMAS-NMR-Based Metabolomics Approach to Discover Key Differences in Cow and Goat Milk Yoghurt Metabolomes

**DOI:** 10.3390/foods13213483

**Published:** 2024-10-30

**Authors:** Sujatha Kandasamy, Won-Seo Park, In-Seon Bae, Jayeon Yoo, Jeonghee Yun, Van-Ba Hoa, Jun-Sang Ham

**Affiliations:** Animal Products Research and Development Division, National Institute of Animal Science, Rural Development Administration, Wanju-gun 55365, Republic of Korea

**Keywords:** cow milk yoghurt, goat milk yoghurt, metabolites, HRMAS-NMR, enrichment analysis

## Abstract

This study highlights the differences in the metabolomes of cow milk yoghurt (CY) and goat milk yoghurt (GY) using a nuclear magnetic resonance (NMR)-based metabolomic approach. The 1H HRMAS-NMR spectrum displayed 21 metabolites comprising organic acids, sugars, amino acids, amino acid derivatives and phospholipids. The orthogonal partial least squares discriminant analysis model clearly separated CY and GY groups, implying differences in metabolite composition. The corresponding Variable Importance in Projection (VIP) plot revealed that choline, sn-glycero-3-phosphocholine, O-phosphocholine, fucose, citrate, sucrose, glucose and lactose mainly contributed to the group separation (VIP > 1). Hierarchical cluster analysis further confirmed the metabolome similarities and differences between CY and GY. Additionally, 12 significantly differential metabolites (with a fold change > 1.5 and *p*-value < 0.05) were identified, with 1 downregulated and 11 upregulated. Pathway impact analysis revealed the correlation of significant metabolites with starch and sucrose metabolism, galactose metabolism, and the citrate cycle. Furthermore, receiver operating characteristic curve analysis identified eight metabolites (choline, sn-glycero-3-phosphocholine, fucose, O-phosphocholine, glucose, citrate, 2-oxoglutarate, lactose and sucrose) as candidate biomarkers. This study represents the first utilization of HRMAS-NMR to analyze the metabolomic profiles of yoghurt made from cow and goat milk. In conclusion, these findings provide preliminary information on how NMR-based metabolomics can discriminate the metabolomes of yoghurt prepared from the milk of two different animals, which may be valuable for authenticity and adulteration assessments.

## 1. Introduction

Yoghurt is widely recognized as a popular fermented dairy product that serves as a healthy food choice in the modern diet. It is highly acceptable by consumers for its rich nutritive content and possible health advantages such as lactose tolerance, enhancement of the immune system, and reduced risk of gastrointestinal disorders and cardiometabolic diseases [1,2]. Yoghurt is currently marketed as “Bio-Yoghurts” to deliver functional ingredients such as prebiotics, probiotics and symbiotics that can offer specific health benefits to the body upon consumption [3,4]. Yoghurt is traditionally produced through fermenting milk using starter cultures (*Lactobacillus delbrueckii* subsp. bulgaricus and *Streptococcus thermophiles*) that produce exometabolites which regulate the acidity, texture, taste, and aroma in the end product [1]. Moreover, innovations in product development lead to the use of multistrain cultures and the availability of diverse flavours and types of yoghurt, significantly increasing its demand and consumption [3,4,5].

Although cow milk dominates the dairy market, the unprecedented demand for goat milk worldwide has attracted interest in developing functional dairy products. In addition to the high nutritive and medicinal values of goat milk, the incidence of cow milk allergies, lactose intolerance and gastrointestinal tract disorders are the reasons for possible alternatives. There are many qualitative differences between cow and goat milk regarding protein content, smaller fat globules, oligosaccharides and medium-chain fatty acids [6,7]. These differences also result in products with different technological properties that intrigued researchers to study the type of metabolites produced during the fermentation of yoghurt [8,9]. Various metabolites, like acids and lipids, that are yielded in the fermentation and storage processes can considerably alter the physicochemical and functional characteristics of yoghurt, such as texture and taste [10,11]. The metabolite production can also vary depending on the type of raw materials, processing methods and culture types used in yoghurt production [12,13,14].

Metabolomics serves as an efficient tool to rapidly detect and quantify smaller molecules in biological matrices using high-throughput techniques. In recent years, metabolomics-based techniques have been extensively utilized on dairy and dairy products to identify potential biomarkers and various adulterants that are directly related to quality, safety and authenticity [8,15,16,17]. Most of these studies have been carried out using liquid chromatography–tandem mass spectrometry (LC-MS) [18] and gas chromatography–mass spectrometry (GC-MS) [8,11,19], which involve extraction and separation of compounds from products before analysis [11,14]. In the aforementioned methods, even basic actions can lead to significant alternations to the original mixture, both qualitatively and quantitatively, in addition to time consumption. The remarkable feature of HRMAS-NMR spectroscopy is to provide rapid and accurate identification of overall metabolites with quantitative information in a single step without any extraction, although the method is expensive. Recently, NMR has been proven to effectively identify a broad spectrum of metabolites in milk [20] and fermented milk products [12,13,21,22]. However, only a few publications focus on comparing the NMR profiles of milk and milk products from different dairy animals.

To date, there is no published literature on the metabolite composition of domestically produced yoghurts in Korea. Specifically, NMR profile data of yoghurt are unavailable or still limited. To the best of our knowledge, this is the first attempt to compare NMR profiles of yoghurt produced from cow and goat milk. In this study, our objective was to utilize 1H HRMAS-NMR spectroscopy to derive the metabolite profiles of yoghurt produced from these two dairy animals and to differentiate between them using a metabolomic approach. Additionally, we attempt to identify the potential metabolic pathways correlated to significant differential metabolites in cow milk yoghurt (CY) compared to goat milk yoghurt (GY), which could help protect their uniqueness and contribute to a deeper understanding of how to improve their technological properties and potential health benefits.

## 2. Materials and Methods

### 2.1. Collection of Yoghurt Samples

A total of 45 samples of domestic yoghurt made from cow (37) and goat (8) milk were collected from native producers across various regions in the Republic of Korea (ROK) for the purpose of this study. These samples were collected in sterile containers and subsequently stored at −20 °C.

### 2.2. Preparation of Samples for NMR Analysis

3-(trimethylsilyl) propionic-2,2,3,3-d4 acid sodium salt (TSP-d4) and deuterium oxide (D2O) were acquired from Sigma-Aldrich Korea Ltd. (Seoul, Republic of Korea). For the NMR analysis, 20 mg of each yoghurt sample was transferred into a 4 mm NMR nanotube (Agilent Technologies, Palo Alto, CA, USA), followed by the addition of 20 μL of D2O solution containing 4 mM TSP-d4 into each tube.

### 2.3. NMR Measurement and Analysis

All spectral data were obtained from a 600 MHz HR-MAS (High Resolution-Magic Angle Spinning) NMR spectrometer outfitted with a 4 mm gHX NanoProbe (Agilent Technologies, Palo Alto, CA, USA). The spinning rate was 2000 Hz, and the PRESAT-CPMG (Carr–Purcell–Meiboom–Gill) pulse sequence was utilized to eliminate the signals from macromolecules and residual water. The sample temperature was held constant at 25 °C (298.15 K) throughout the experiment. Each spectrum was recorded with a 3.0 s acquisition time, 90° pulse length with 7.075 μs, and a 3.0 s relaxation delay along with 128 transients, resulting in an overall acquisition time of 13 min 9 s per sample. The resulting free induction decays underwent a 0.5 Hz exponential multiplication line-broadening factor before Fourier transformation.

### 2.4. Identification and Quantification of Metabolites

Metabolite identification and quantification in each sample were performed using the Chenomx Profiler and 600 MHz library database in Chenomx NMR Suite 7.1 (Chenomx Inc., Edmonton, AB, Canada), respectively. Individual metabolite concentration was determined by referencing a known concentration of TSP-d4. All spectra were processed by conversion to the frequency domain and automatic phase, baseline-corrected, and referenced with TSP-d4 resonance at 0.00 ppm.

### 2.5. Multivariate Statistical Analysis

The data obtained after metabolite annotations were uploaded to the MetaboAnalyst 6.0 web server (https://www.metaboanalyst.ca; accessed on 6 August 2024) [23]. Before conducting multivariate statistical analysis, the data underwent filtering and preprocessing steps (missing value estimation, log transformation and Pareto scaling). Pareto scaling is commonly used for datasets with large dynamic variations. This scaling method closely preserves the original measurements and improves the pattern recognition of metabolite data by tailoring sensitivity reduction [23]. Chemometric tools like principal component analysis (PCA) and orthogonal partial least squares discriminant analysis (OPLS-DA) were used to identify the similarities or differences between the groups. The contribution of variables in the group separation was characterized using S-plot and Variable Influence in Projection (VIP) scores. Further, the OPLS-DA model was cross-validated using R2 (interpretability) and Q2 (predictability) parameters from a permutation test containing 1000 iterations. A heat map was generated using hierarchical clustering analysis (HCA) to illustrate the quantitative relationships between the metabolic profiles of each sample. Volcano plots were utilized to compare the metabolite concentrations between CY and GY groups to identify significant differential metabolites by combining with fold change (>1.5) and *p*-values (<0.05). Using the pathway analysis module in Metaboanalyst 6.0, the biochemical pathways for all significant metabolites were mapped with the KEGG compound database (http://www.genome.jp/kegg/; accessed on 17 August 2024). Finally, metabolites with AUC values > 0.7 in the receiver operating characteristic (ROC) curve from the biomarker analysis module were considered potential biomarkers.

## 3. Results and Discussion

### 3.1. ^1^H HRMAS-NMR Spectra and Metabolic Assignments of Yoghurt

**Figure 1:**
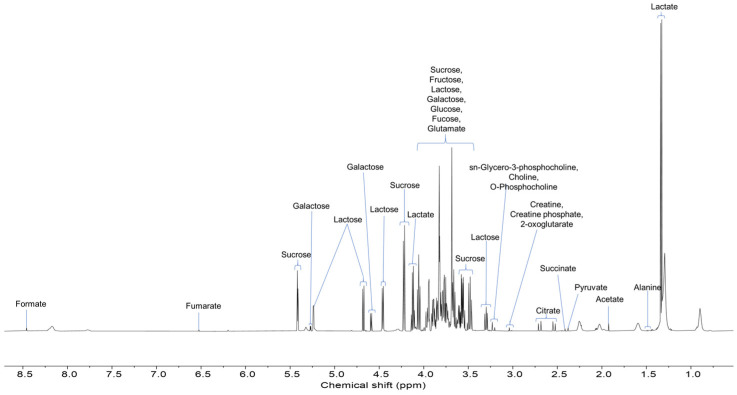
Results of 600 MHz ^1^H-NMR HRMAS spectrum and metabolite annotation of a yoghurt sample.

Yoghurt is recognized as a nutrient-dense food rich in available protein, amino acids, fatty acids, lipids, minerals, organic acids, sugars, and volatile aroma compounds that contribute to the overall flavour and taste profile [1,10]. In our study, ^1^H HRMAS-NMR detected metabolites from domestic cow and goat yoghurt samples, and the resultant NMR spectrum is depicted in Figure 1. Detailed information on chemical shifts and peak multiplicity are outlined in Table 1. We identified a total of 21 metabolites comprising nine organic acids (2-oxoglutarate, acetate, citrate, creatine phosphate, formate, fumarate, lactate, pyruvate, and succinate), six sugars (fructose, fucose, galactose, glucose, lactose, and sucrose), two amino acids (alanine and glutamate), two amino acid derivatives (choline and creatine), and two phospholipids (o-phosphocholine and sn-glycero-3-phosphocholine). These findings are consistent with the listed metabolites from yoghurt reported by Lu et al. [10] using the HRMAS-NMR technique. The identification and characterization of several metabolites from different classes have provided an in-depth understanding of the yoghurt composition. A prior study that utilized GC–MS methodology to compare yoghurt from cow and goat milk revealed the presence of metabolites comprising simple sugars, amino acids, organic acids and fatty acid derivatives [8].

### 3.2. Multivariate Analysis to Discriminate Cow Milk and Goat Milk Yoghurt

The quality of processed dairy foods relies on the physicochemical and technological properties of milk. It is well established that milk metabolites vary significantly among different ruminant animals. Therefore, a metabolomics approach utilizing ^1^H-NMR was employed to differentiate the major metabolite profiles of yoghurt derived from cow and goat milk.

**Figure 2:**
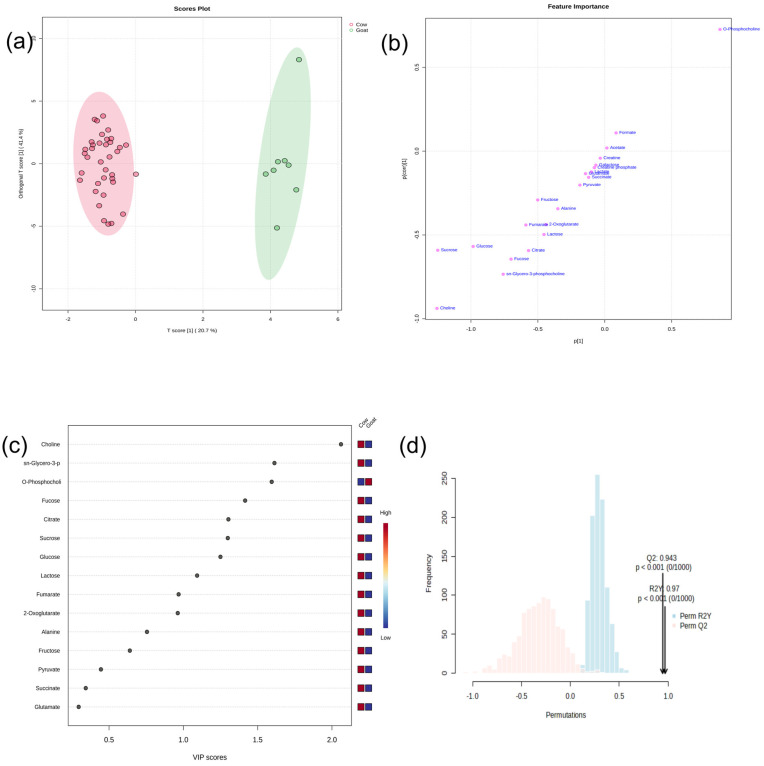
OPLS-DA model for metabolic profiles in yoghurt samples from cow milk (CY) versus goat milk (GY). (**a**) Score plot, (**b**) S-plot, (**c**) VIP plot, and (**d**) permutation test plots of the OPLS-DA model.

Principal component analysis (PCA) and orthogonal partial least squares discriminant analysis (OPLS-DA) are widely used multivariate tools for the identification of significant variations between metabolic samples. Initially, an untargeted PCA is employed to visualize the overall distribution of samples under study, including outliers. The resulting score plot showed a slight overlap between the two groups (indicating that they share similar metabolite profiles) along PC1 and PC2, accounting for 50% and 18.6% of the variance (Figure S1), respectively. No outliers were found, and all samples were within the eclipse, clearly indicating that the separation was based on the metabolite composition between the yoghurt samples and the data deserve further study. Additionally, a pairwise OPLS-DA was conducted to explore the specific differences between the groups. The outcomes of the discriminating analysis are illustrated in Figure 2. As seen in the subsequent score plot (Figure 2a), the two groups exhibited clear separation by locating distinctly on opposite sides of the ellipse, substantiating greater differences in their metabolomes. The first and second main components contributed to 20.7% and 41.4% of the entire dataset’s variability, respectively.

Moreover, a corresponding S-plot (Figure 2b) derived from the OPLS-DA model highlighted the metabolites responsible for separation between the groups. In the S-plot, each dot represents an individual metabolite. Metabolites with higher values of *p* and p(corr) are generally found on the upper far right and lower far left quadrants and are most relevant for group discrimination [24]. In agreement, higher O-phosphocholine in GY samples was observed in the upper right of the plot, while higher choline, sucrose and glucose in CY samples were in the lower left.

In addition, the Variable Importance in Projection (VIP) plot highlights the significant contribution of each metabolite, ranking them based on their VIP scores from highest to lowest. The top 15 metabolites were selected for a meaningful interpretation with a focus on those with VIP > 1 that are most relevant in explaining the model [25]. From our VIP plot, we identified nine important metabolites, namely choline, sn-glycero-3-phosphocholine, O-phosphocholine, fucose, citrate, sucrose, glucose and lactose, which significantly contributed to the group separation based on their VIP > 1 (Figure 2c). More precisely, the levels were higher for choline, sn-glycero-3-phosphocholine, fucose, citrate, sucrose, glucose and lactose, and lower for O-phosphocholine in CY compared to GY. Glycerophosphocholine is associated with choline biosynthesis and serves as an intermediate product in phosphatidylcholine metabolism [8]. Studies have suggested that glycerophosphocholine supplementation can improve learning and memory performance while reducing the risk of cardiovascular diseases [26]. Fucose is a monosaccharide that supports the growth of bifidobacteria in newborns, shields intestinal mucosa cells from pathogens, and contributes to neonatal brain development [27].

Finally, permutation tests (Figure 2d) were carried out to ascertain the statistical significance of the separation in OPLS-DA. The model’s reliability and stability were defined by R2Y (goodness of fit) ≤ 1 and Q2 (predictive ability) values ≥ 0.4. The R2Y and Q2 values obtained at 1000 permutations (Figure 2d) exceeded 0.9 (R2Y = 0.97 and Q2 = 0.943), indicating the model has higher accuracy and predictable levels [11].

**Figure 3:**
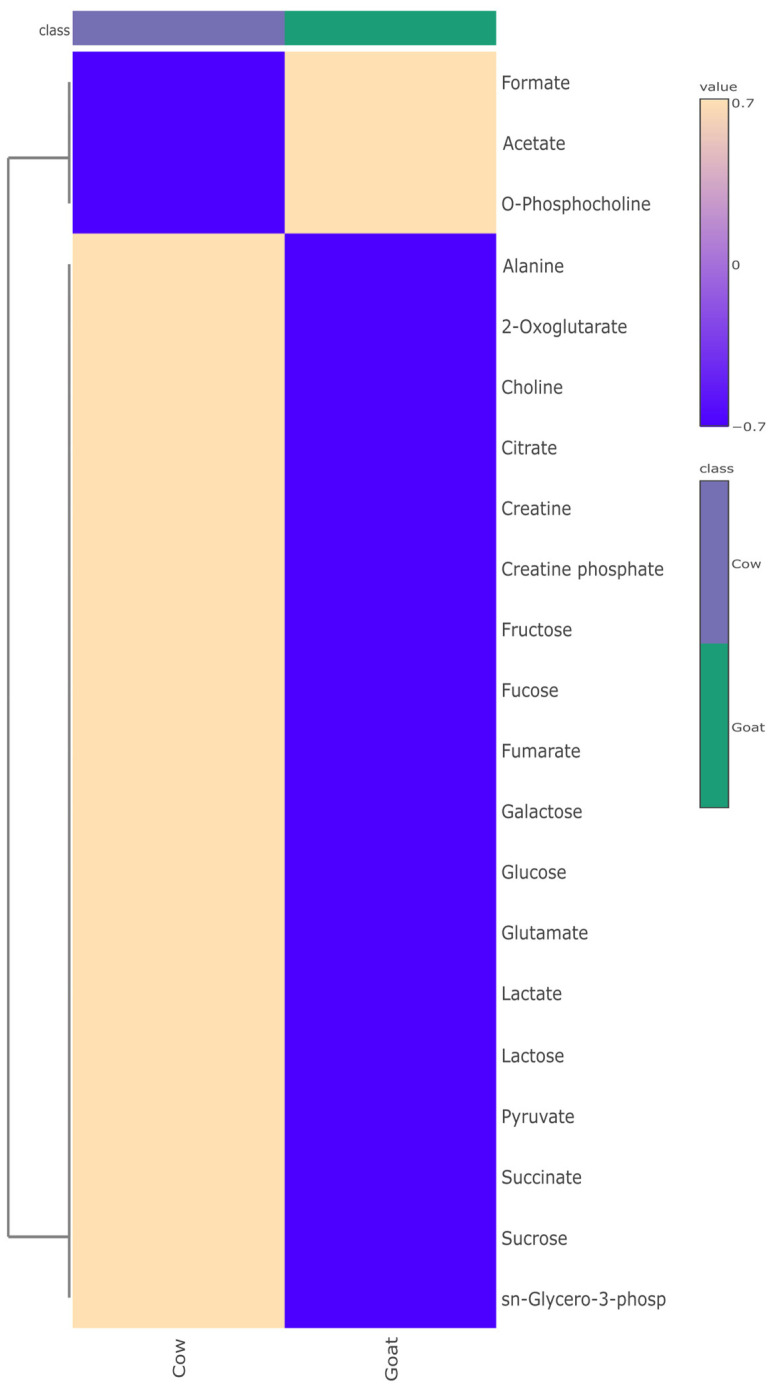
Hierarchical clustered heat map of the metabolite levels in samples of cow milk yoghurt vs. goat milk yoghurt.

Using hierarchical cluster analysis (HCA), we decided to study the similarities and differences in metabolite profiles within yoghurt samples, along with the variations in metabolite composition between the two groups. Metabolites from CY and GY samples from this study were categorized and visualized in heat maps based on the Euclidean distance matrix of quantitative values (Figure 3). The colour gradient from beige to violet in the heat maps illustrates metabolite concentrations. The dendrogram in Figure 3 shows that CY and GY samples are grouped distinctly into separate classes based on significant correlation. On the other hand, two clades were observed in the metabolite clustering. The upper clade exhibited a higher accumulation of formate, acetate and O-phosphocholine in GY, while the remaining metabolites showed higher accumulation in CY. These findings indicated that the yoghurt samples within each group were closely related to each other and differed significantly between the groups, especially in terms of the top 15 differential metabolites.

### 3.3. Screening for Differential Metabolites in Yoghurt

To better understand the differences in metabolite concentrations between CY and GY samples, metabolites exhibiting a fold change (FC) of >1.5 and a *p*-value of <0.05 were identified and visualized through volcano plots. In total, we identified 12 significant differential metabolites, of which 1 (O-phosphocholine) was downregulated and 11 (choline, sn-glycero-3-phosphocholine, fucose, citrate, glucose, sucrose, lactose, fumarate, 2-oxoglutarate, fructose and alanine) were upregulated in the comparison of CY vs. GY (Table 2). A prior study by Sharma et al. [8] revealed 21 differential metabolites between cow and goat milk yoghurt by the GC-MS approach. Similarly to our findings, an upregulation of sn-glycero-3-phosphocholine was found between the CY and GY samples. The changes in the relative concentration of the differential metabolites are illustrated as box-and-whisker plots in Figure S2. Among the differential metabolites, choline, fucose, 2-oxoglutarate, glucose, fructose and alanine were present in relatively higher concentrations, while O-phosphocholine, sucrose and fumarate were found at lower concentrations in samples of CY than GY. The increased amount of saccharides found in yoghurt can be related to the extracellular polysaccharides (glucans or polymers that include glucose, galactose and fructose as constituent sugars) produced by *L. delbrueckii* and *S. thermophiles* [28].

### 3.4. Pathway Analysis

**Figure 4:**
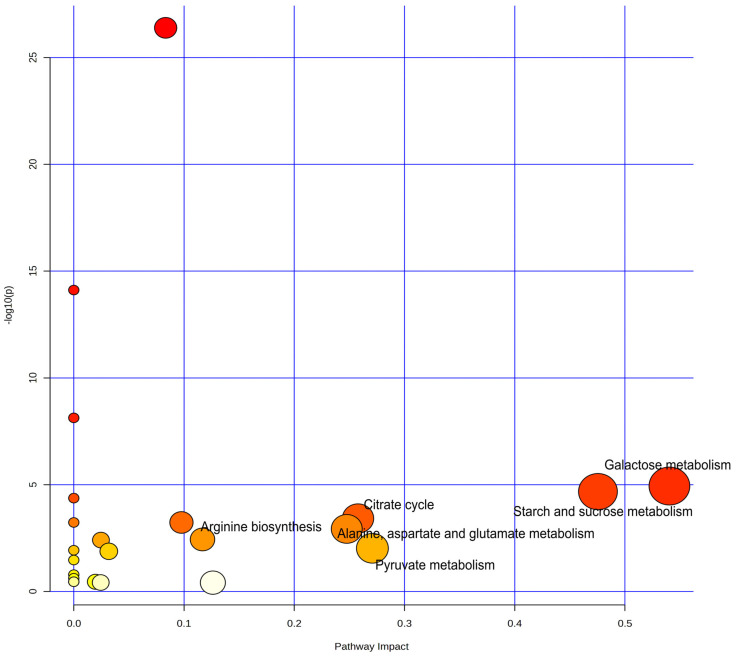
Metabolic pathways of significantly different metabolites in cow milk yoghurt vs. goat milk yoghurt. In the plot, circles denote the matched metabolic pathways, while the horizontal and vertical coordinates denote the pathway impact value and −log10(*p*) value, respectively. The colour gradient and size of circles were based on *p*-values (yellow—high *p*-value; red—low *p*-value) and pathway impact scores (bigger circles denote greater impact score), respectively.

The process of converting lactose in milk into lactic acid by the starter culture is complex and involves numerous reactions that result in several intermediates in metabolic pathways. These pathways reduce the pH and bring desirable biochemical changes in yoghurt production [21]. Pathway analysis has emerged as one of the most widely used approaches for the functional interpretation of metabolomics data. The pathways of significant differential metabolites in CY and GY were mapped using the KEGG metabolite database. In Figure 4, each circle denotes a unique metabolic pathway. The horizontal position and circle size indicate the pathway’s impact, while the vertical position and circle colour indicate the negative log of *p*-values in the enrichment assay. The size and colour of each circle are correlated with the pathway impact values and *p*-values, respectively. As the *p*-value decreases, the circles become redder. The larger and deeper-coloured circles correspond to the most substantial metabolic pathways.

The results of enrichment analysis conducted on the MetaboAnalyst 6.0 platform revealed 16 statistical pathways covering a broad range of metabolic pathways (Table 3). Pathways exhibiting a raw *p*-value of <0.05 and an impact value of >0.1 were classified as potentially disturbed pathways [29]. Based on these criteria, three significantly enriched metabolic pathways (Figure S3) were identified: starch and sucrose metabolism (raw *p*-value < 0.01; 0.476 impact score), galactose metabolism (raw *p*-value < 0.01; 0.184 impact score) and citrate cycle (raw *p*-value < 0.01; 0.179 impact score). These pathways play a major and essential role in producing bioactive compounds during yoghurt fermentation. Galactose metabolism denotes the enzymatic conversion of lactose in milk (pH 6.7) to lactate by the starters, through the glycolytic pathway and fermentation of lactic acid. Additionally, galactose can be produced through the degradation of galactooligosaccharides. Citrate, a key component of the tricarboxylic acid (TCA) cycle, is derived from glucose via the glycolytic pathway and through the oxidative conversion of pyruvate into acetyl-CoA by pyruvate dehydrogenase. Furthermore, the starter LAB can convert citrate into aroma compounds (diacetyl, acetaldehyde), thereby enhancing the food quality in fermented products [21]. These findings provide valuable insights into the fermentation of yoghurts made from cow milk and goat milk.

The pathway map of metabolic enrichment highlights the most substantial circles assigned to starch and sucrose metabolism, and galactose metabolism, which are crucial in yoghurt production. Metabolites, namely sucrose, D-galactose, lactose, D-fructose and D-glucose, were found to be involved in galactose metabolism. The metabolites D-fructose, sucrose and D-glucose can also be hydrolyzed during starch and sucrose metabolism and can then enter into the citrate cycle via a glycolytic pathway, contributing to the production of several metabolites that enhance the quality of the end product. These results conclude that specific metabolites regulate pathways to achieve the desired traits in yoghurt.

Further, by employing KEGG databases, we mapped 12 key metabolites (Table 3): 2-oxoglutarate, fumarate, D-fructose, D-glucose, citrate, choline, sn-Glycero-3-phosphocholine, creatine, pyruvate, sucrose, lactose and L-alanine. Notably, 2-oxoglutarate and fumarate were noticed to be included in five metabolic pathways, D-fructose in four pathways, and D-glucose and citrate in three pathways. Furthermore, choline and sn-glycero-3-phosphocholine were associated with two pathways. These results confirm that the differential metabolites intersected multiple pathways, indicating their significant impact on the overall pathway network.

### 3.5. Selection of Candidate Biomarkers

**Figure 5:**
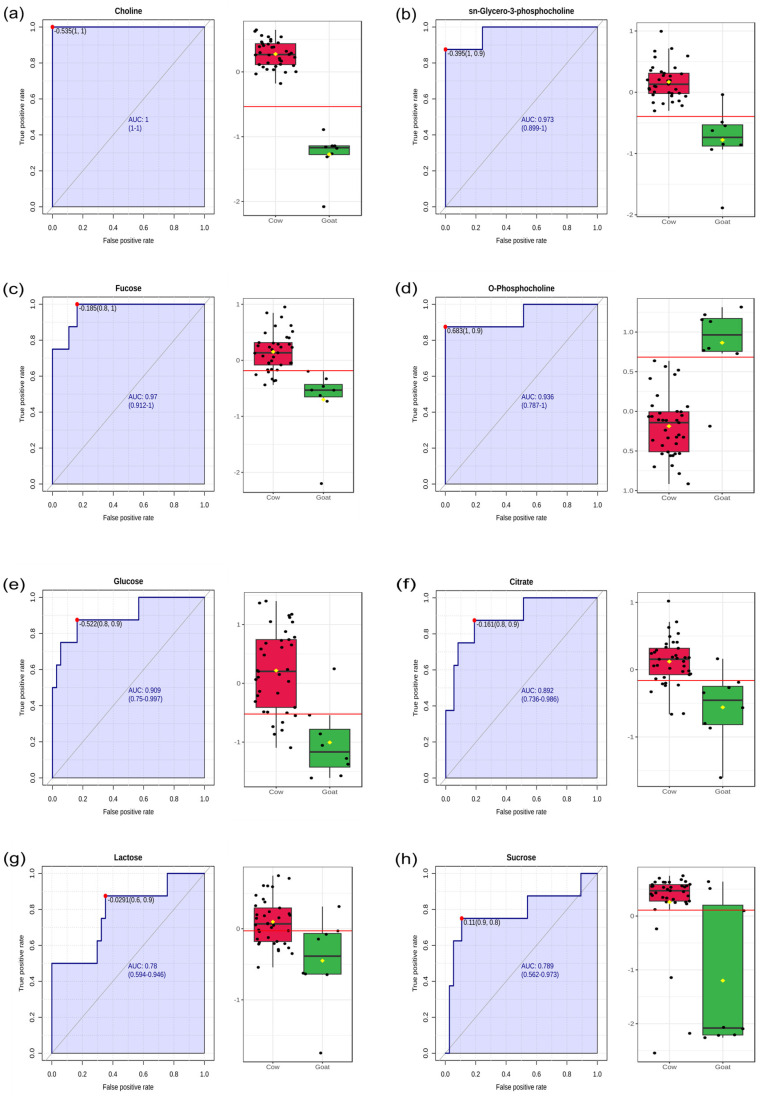
Selection of biomarkers by HRMAS-NMR-based metabolomics. Top-ranked metabolites (**a**–**h**) based on area under the ROC curve (AUC) values in discrimination of cow milk yoghurt vs. goat milk yoghurt.

Generally, the VIP scores from the OPLS-DA model with high accuracy and predictability (R2 and Q2 values) were utilized as a basis for the selection of biomarkers. However, the model was found to lack transparency and was insufficient for selecting biomarkers [30]. Therefore, we employed receiver operating characteristic (ROC) curve analysis to discover biomarkers among the significant differential metabolites. The areas under the ROC curves (AUCs) were used to determine the effectiveness of potential metabolites. According to Xia et al. [30], AUC values can be interpreted as follows: 0.9–1.0 = excellent; 0.8–0.9 = good; 0.7–0.8 = fair; 0.6–0.7 = poor; 0.5–0.6 = fail. Our AUC analysis (Figure 5) revealed that only one metabolite, choline, had an AUC value of 1. Choline has been revealed as a potential biomarker in earlier studies for distinguishing milk from different dairy animals [15,31]. Three metabolites were found with AUC values of >0.9 (sn-glycero-3-phosphocholine, fucose and O-phosphocholine) and >0.8 (glucose, citrate and 2-oxoglutarate). Two metabolites (lactose and sucrose) had AUC values > 0.7. Hence, we can conclude that the eight metabolites with AUC values > 0.7 indicate strong discriminatory ability and potential utility as robust biomarkers for discriminating between CY and GY samples.

## 4. Conclusions

In this study, we utilized a combined HRMAS-NMR and metabolomics approach to analyze the variation in metabolite composition in cow and goat milk yoghurt from several dairy farms in the ROK. From the NMR spectrum, we identified 21 metabolites comprising nine organic acids, six sugars, two amino acids, two amino acid derivatives, and two phospholipids. A combination of NMR and metabolomics tools enabled the identification of significant metabolites between the cow and goat milk yoghurt. Unlike the PCA model, OPLS-DA exhibited an evident separation between the two groups. The corresponding S-plot explained that group separation was due to higher levels of O-phosphocholine in GY, while CY contained higher levels of choline, sucrose and glucose. Additionally, the VIP plot revealed nine important metabolites (VIP > 1), namely choline, sn-glycero-3-phosphocholine, O-phosphocholine, fucose, citrate, sucrose, glucose and lactose, which significantly contributed to the group differentiation. By employing fold change criteria (>1.5) and a *p*-value < 0.05 threshold in the volcano plot, 12 significant differential metabolites were identified between cow and goat milk yoghurt. Pathway impact analysis revealed that starch and sucrose metabolism, as well as galactose metabolism, played major roles in the yoghurt production process. Understanding these biochemical alterations could help us to understand the species-specific differences and their impact on yoghurt composition. Through ROC curve analysis, we selected eight metabolites (AUC values > 0.7) as potential candidate biomarkers for differentiating the cow and goat milk yoghurt. Further exploration of variables such as time–temperature combinations during processing and/or storage, starter culture types, and the addition of active ingredients (probiotics/prebiotics) could provide in-depth insights into metabolite regulation in yoghurt. Although our study did not consider the association of metabolite changes with physicochemical properties, the promising results encourage future studies in this direction.

## Figures and Tables

**Table 1:** List of metabolites identified and their respective ^13^C chemical shifts in cow milk yoghurt and goat milk yoghurt samples using ^1^H HRMAS-NMR.

Metabolite	Chemical Shift (ppm) ^a^
2-Oxoglutarate	3.0 (t), 2.4 (t)
Acetate	1.9 (s)
Alanine	3.8 (m), 1.5 (d)
Choline	4.1 (m), 3.5 (m), 3.2 (s)
Citrate	2.7 (d), 2.5 (d)
Creatine	3.9 (s), 3.0 (s)
Creatine phosphate	3.9 (s), 3.0 (s)
Formate	8.4 (s)
Fructose	4.1–3.5 (m)
Fucose	5.3 (d), 5.2 (dd), 4.6 (d), 3.8 (m), 1.2 (dd)
Fumarate	6.5 (s)
Galactose	5.3 (d), 4.6 (d), 3.8 (m)
Glucose	5.2 (d), 4.6 (d), 3.7 (m)
Glutamate	3.7 (q), 2.3 (m)
Lactate	4.1 (q), 1.3 (d)
Lactose	5.2 (d), 4.7 (d), 4.4 (m), 3.8 (m), 3.3 (t)
O-Phosphocholine	4.2 (m), 3.6 (m), 3.2 (s)
Pyruvate	2.4 (s)
succinate	2.4 (s)
sucrose	5.4 (d), 4.2 (d), 3.8 (m)
sn-Glycero-3-phosphocholine	3.9 (m), 3.2 (s)

^a^ Peak multiplicities are denoted as (s)—singlet; (m)—multiplet; (d)—doublet; (t)—triplet; and (q)—quartet.

**Table 2:** List of metabolites that were upregulated and downregulated in yoghurt samples prepared from cow milk compared to goat milk.

Metabolites	*p*-Value	Fold Change	Regulation	VIP
Choline	1.38 × 10^−20^	3.227	Upregulated	2.061
sn-Glycero-3-phosphocholine	7.40 × 10^−9^	1.444	Upregulated	1.613
O-Phosphocholine	3.43 × 10^−8^	−1.995	Downregulated	1.594
Fucose	3.13 × 10^−6^	1.374	Upregulated	1.415
Citrate	4.23 × 10^−5^	0.953	Upregulated	1.303
Glucose	4.26 × 10^−5^	3.339	Upregulated	1.249
Sucrose	7.05 × 10^−5^	1.361	Upregulated	1.299
Lactose	0.00079	0.661	Upregulated	1.092
Fumarate	0.00284	0.78	Upregulated	0.968
2-Oxoglutarate	0.00403	0.752	Upregulated	0.962
Fructose	0.03486	2.764	Upregulated	0.640
Alanine	0.03550	0.676	Upregulated	0.755

**Table 3:** List of the metabolic pathways altered in yoghurt samples prepared from cow milk yoghurt in comparison to goat milk yoghurt.

Pathway6	Total Cmpd	Counts	Raw *p*-Value	−log10(*p*)	Holm Adjust	False Discovery Rate	Impact Score	Metabolites Involved
Glycerophospholipid metabolism	36	3	4.08 × 10^−27^	26.390	6.52 × 10^−26^	6.52 × 10^−26^	0.083	Choline phosphate; choline; sn-Glycero-3-phosphocholine
Glycine, serine and threonine metabolism	34	1	1.38 × 10^−20^	19.861	2.07 × 10^−19^	1.10 × 10^−19^	0.000	Choline; creatine; pyruvate
Ether lipid metabolism	20	1	7.40 × 10^−9^	8.131	1.04 × 10^−7^	3.95 × 10^−8^	0.000	sn-Glycero-3-phosphocholine
Galactose metabolism	27	4	1.11 × 10^−5^	4.954	0.0001	4.45 × 10^−5^	0.184	Sucrose; lactose; D-fructose; D-glucose
Starch and sucrose metabolism	18	3	2.10 × 10^−5^	4.677	0.0003	6.73 × 10^−5^	0.476	D-fructose; sucrose; D-glucose
Glyoxylate and dicarboxylate metabolism	32	1	4.23 × 10^−5^	4.374	0.0005	9.75 × 10^−5^	0.032	Citrate
Neomycin, kanamycin and gentamicin biosynthesis	2	1	4.26 × 10^−5^	4.370	0.0005	9.75 × 10^−5^	0.000	D-glucose
Citrate cycle (TCA cycle)	20	3	0.000	3.728	0.002	0.000	0.179	2-oxoglutarate; citrate; fumarate
Alanine, aspartate and glutamate metabolism	28	3	0.000	3.728	0.002	0.000	0.050	L-alanine; citrate; fumarate; 2-oxoglutarate
Fructose and mannose metabolism	20	2	0.001	3.232	0.004	0.001	0.098	D-fructose; 6-Deoxy-L-galactose
Amino sugar and nucleotide sugar metabolism	42	2	0.001	3.232	0.004	0.001	0.000	6-Deoxy-L-galactose; D-fructose
Arginine biosynthesis	14	2	0.001	2.874	0.007	0.002	0.000	2-oxoglutarate; fumarate
Tyrosine metabolism	42	1	0.003	2.546	0.011	0.003	0.025	Fumarate
Pyruvate metabolism	23	1	0.003	2.546	0.011	0.003	0.000	Fumarate
Butanoate metabolism	15	1	0.004	2.395	0.011	0.004	0.000	2-oxoglutarate
Lipoic acid metabolism	28	1	0.004	2.395	0.011	0.004	0.000	2-oxoglutarate

## Data Availability

The original contributions presented in the study are included in the article/Supplementary Material, further inquiries can be directed to the corresponding author.

## Supplementary Materials

The following supporting information can be downloaded at https://www.mdpi.com/article/10.3390/foods13213483/s1: Figure S1: PCA score plot displaying the metabolic variation in yoghurt samples prepared from cow milk and goat milk. Figure S2: Box–whisker plots showing normalized levels of significantly differential metabolites in yoghurt samples from cow and goat milk. Boxes denote the interquartile range (IQR) between the bottom and top boundaries (25th and 75th percentiles); the horizontal line inside the box denotes the median. The lower and upper whiskers are the 5th and 95th percentiles, respectively. Figure S3: Snapshot of KEGG pathway maps for (a) starch and sucrose metabolism, (b) galactose metabolism, and (c) citrate cycle significantly altered in the samples of cow milk and goat milk yoghurt.

## References

[1] Aryana K.J., Olson D.W. (2017). A 100-Year Review: Yogurt and Other Cultured Dairy Products. J. Dairy Sci..

[2] Fernandez M.A., Fisberg M., Marette A. (2017). Role of Yogurt in the Nutrition and Health of Children and Adolescents. Yogurt in Health and Disease Prevention.

[3] Fazilah N.F., Ariff A.B., Khayat M.E., Rios-Solis L., Halim M. (2018). Influence of Probiotics, Prebiotics, Synbiotics and Bioactive Phytochemicals on the Formulation of Functional Yogurt. J. Funct. Foods.

[4] Bankole A.O., Irondi E.A., Awoyale W., Ajani E.O. (2023). Application of Natural and Modified Additives in Yogurt Formulation: Types, Production, and Rheological and Nutraceutical Benefits. Front. Nutr..

[5] Wang J., Zhao W., Guo S., Sun Y., Yao K., Liu Z., Sun Z., Kwok L.Y., Peng C. (2021). Different Growth Behaviors and Metabolomic Profiles in Yogurts Induced by Multistrain Probiotics of Lactobacillus Casei Zhang and Bifidobacterium Lactis V9 under Different Fermentation Temperatures. J. Dairy Sci..

[6] Guha S., Sharma H., Deshwal G.K., Rao P.S. (2021). A Comprehensive Review on Bioactive Peptides Derived from Milk and Milk Products of Minor Dairy Species. Food Prod. Process. Nutr..

[7] Chen L., Bagnicka E., Chen H., Shu G. (2023). Health Potential of Fermented Goat Dairy Products: Composition Comparison with Fermented Cow Milk, Probiotics Selection, Health Benefits and Mechanisms. Food Funct..

[8] Sharma H., El Rassi G.D., Lathrop A., Dobreva V.B., Belem T.S., Ramanathan R. (2021). Comparative Analysis of Metabolites in Cow and Goat Milk Yoghurt Using GC–MS Based Untargeted Metabolomics. Int. Dairy J..

[9] Wang L., Wu T., Zhang Y., Yang K., He Y., Deng K., Liang C., Gu Y. (2023). Comparative Studies on the Nutritional and Physicochemical Properties of Yoghurts from Cows’, Goats’, and Camels’ Milk Powder. Int. Dairy J..

[10] Lu Y., Hu F., Miyakawa T., Tanokura M. (2016). Complex Mixture Analysis of Organic Compounds in Yogurt by NMR Spectroscopy. Metabolites.

[11] Sharma H., Ramanathan R. (2021). Gas Chromatography-Mass Spectrometry Based Metabolomic Approach to Investigate the Changes in Goat Milk Yoghurt during Storage. Food Res. Int..

[12] Settachaimongkon S., Nout M.J.R., Antunes Fernandes E.C., van Hooijdonk T.C.M., Zwietering M.H., Smid E.J., Van Valenberg H.J.F. (2014). The Impact of Selected Strains of Probiotic Bacteria on Metabolite Formation in Set Yoghurt. Int. Dairy J..

[13] Trimigno A., Lyndgaard C.B., Atladóttir G.A., Aru V., Engelsen S.B., Clemmensen L.K.H. (2020). An NMR Metabolomics Approach to Investigate Factors Affecting the Yoghurt Fermentation Process and Quality. Metabolites.

[14] Sharma H., Ramanathan R. (2023). GC–MS-Based Metabolomics Approach Reveals Metabolic Variations between Probiotics Incorporated Cow and Goat Milk Yoghurt. Int. J. Dairy Technol..

[15] Yang Y., Zheng N., Zhao X., Zhang Y., Han R., Yang J., Zhao S., Li S., Guo T., Zang C. (2016). Metabolomic Biomarkers Identify Differences in Milk Produced by Holstein Cows and Other Minor Dairy Animals. J. Proteom..

[16] Gu Y., Li X., Chen H., Guan K., Qi X., Yang L., Ma Y. (2021). Evaluation of FAAs and FFAs in Yogurts Fermented with Different Starter Cultures during Storage. J. Food Compos. Anal..

[17] Rehman H., Saipriya K., Singh A.K., Singh R., Meena G.S., Khetra Y., Sharma H. (2024). A Metabolomics Approach to Establish the Relationship between the Techno-Functional Properties and Metabolome of Indian Goat Yoghurt. Foods.

[18] Wu R., Chen J., Zhang L., Wang X., Yang Y., Ren X. (2021). LC/MS-Based Metabolomics to Evaluate the Milk Composition of Human, Horse, Goat and Cow from China. Eur. Food Res. Technol..

[19] Scano P., Murgia A., Pirisi F.M., Caboni P. (2014). A Gas Chromatography-Mass Spectrometry-Based Metabolomic Approach for the Characterization of Goat Milk Compared with Cow Milk. J. Dairy Sci..

[20] Tenori L., Santucci C., Meoni G., Morrocchi V., Matteucci G., Luchinat C. (2018). NMR Metabolomic Fingerprinting Distinguishes Milk from Different Farms. Food Res. Int..

[21] Lu Y., Ishikawa H., Kwon Y., Hu F., Miyakawa T., Tanokura M. (2018). Real-Time Monitoring of Chemical Changes in Three Kinds of Fermented Milk Products during Fermentation Using Quantitative Difference Nuclear Magnetic Resonance Spectroscopy. J. Agric. Food Chem..

[22] Kandasamy S., Yoo J., Yun J., Kang H.B., Seol K.H., Ham J.S. (2020). 1H HRMAS-NMR Based Metabolic Fingerprints for Discrimination of Cheeses Based on Sensory Qualities. Saudi J. Biol. Sci..

[23] Pang Z., Lu Y., Zhou G., Hui F., Xu L., Viau C., Spigelman A.F., Macdonald P.E., Wishart D.S., Li S. (2024). MetaboAnalyst 6.0: Towards a Unified Platform for Metabolomics Data Processing, Analysis and Interpretation. Nucleic Acids Res..

[24] Peng C., Yao G., Sun Y., Guo S., Wang J., Mu X., Sun Z., Zhang H. (2021). Comparative Effects of the Single and Binary Probiotics of Lacticaseibacillus Casei Zhang and Bifidobacterium Lactis V9 on the Growth and Metabolomic Profiles in Yogurts. Food Res. Int..

[25] Nkosi N.J., Shoko T., Manhivi V.E., Slabbert R.M., Sultanbawa Y., Sivakumar D. (2022). Metabolomic and Chemometric Profiles of Ten Southern African Indigenous Fruits. Food Chem..

[26] Syme C., Czajkowski S., Shin J., Abrahamowicz M., Leonard G., Perron M., Richer L., Veillette S., Gaudet D., Strug L. (2016). Glycerophosphocholine Metabolites and Cardiovascular Disease Risk Factors in Adolescents: A Cohort Study. Circulation.

[27] Raynal-Ljutovac K., Lagriffoul G., Paccard P., Guillet I., Chilliard Y. (2008). Composition of Goat and Sheep Milk Products: An Update. Small Rumin. Res..

[28] Zeidan A.A., Poulsen V.K., Janzen T., Buldo P., Derkx P.M.F., Øregaard G., Neves A.R. (2017). Polysaccharide Production by Lactic Acid Bacteria: From Genes to Industrial Applications. FEMS Microbiol. Rev..

[29] Chen Y., Ma Z., Zhong J., Li L., Min L., Xu L., Li H., Zhang J., Wu W., Dai L. (2018). Simultaneous Quantification of Serum Monounsaturated and Polyunsaturated Phosphatidylcholines as Potential Biomarkers for Diagnosing Non-Small Cell Lung Cancer. Sci. Rep..

[30] Xia J., Broadhurst D.I., Wilson M., Wishart D.S. (2013). Translational Biomarker Discovery in Clinical Metabolomics: An Introductory Tutorial. Metabolomics.

[31] Sundekilde U.K., Frederiksen P.D., Clausen M.R., Larsen L.B., Bertram H.C. (2011). Relationship between the Metabolite Profile and Technological Properties of Bovine Milk from Two Dairy Breeds Elucidated by NMR-Based Metabolomics. J. Agric. Food Chem..

