# Supplementary material for: HRMAS-NMR-Based Metabolomics Approach to Discover Key Differences in Cow and Goat Milk Yoghurt Metabolomes
Source: Foods. 2024 Oct 30;13(21):3483. doi: 10.3390/foods13213483 (PMC11545400; doi:10.3390/foods13213483)
Supplement: Supplementary file 1 [file foods-13-03483-s001.zip › foods-3261298-supplementary.pdf]

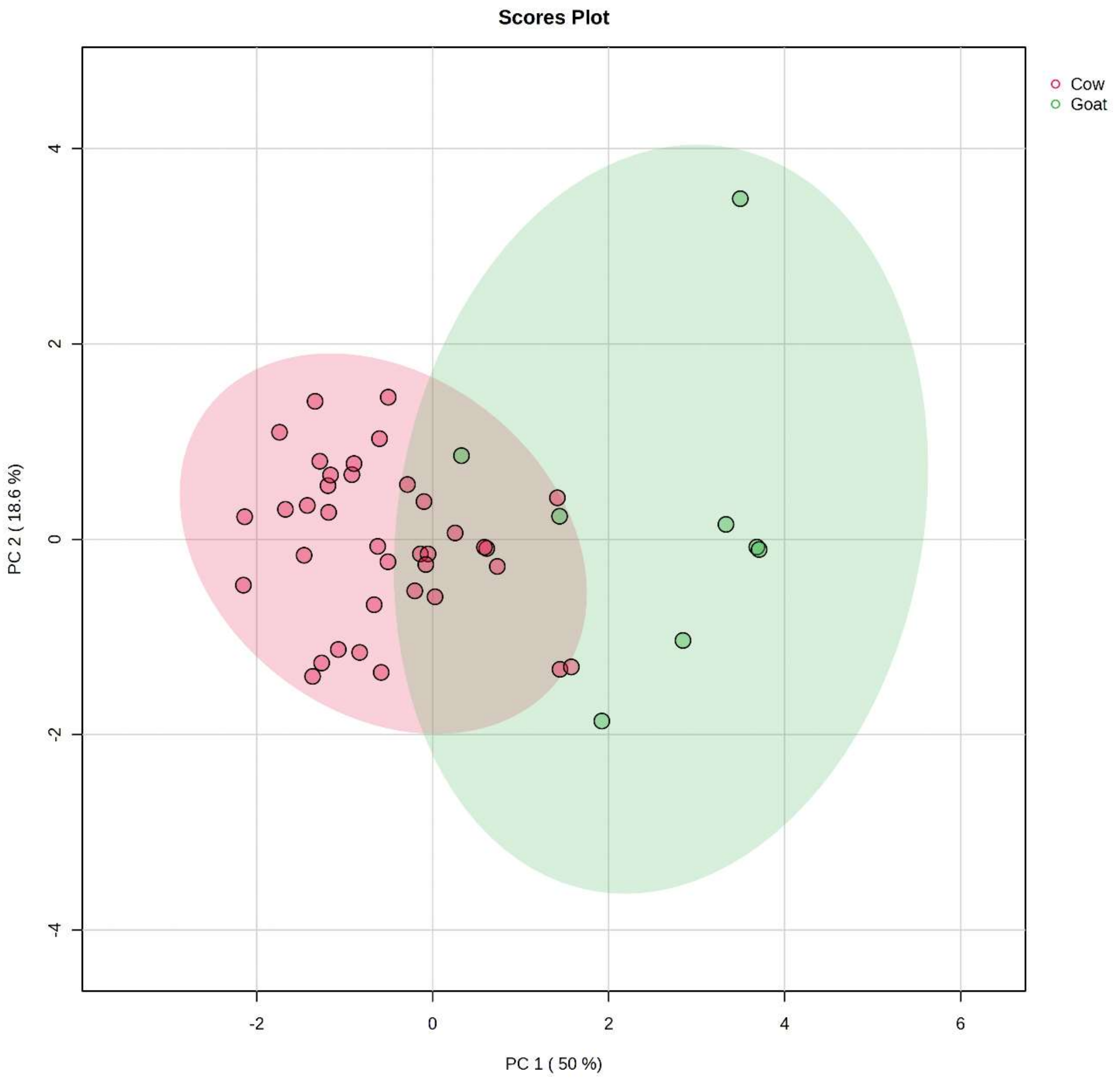

Figure S1: PCA score plot displaying the metabolic variation in yoghurt samples prepared from cow milk and goat milk.

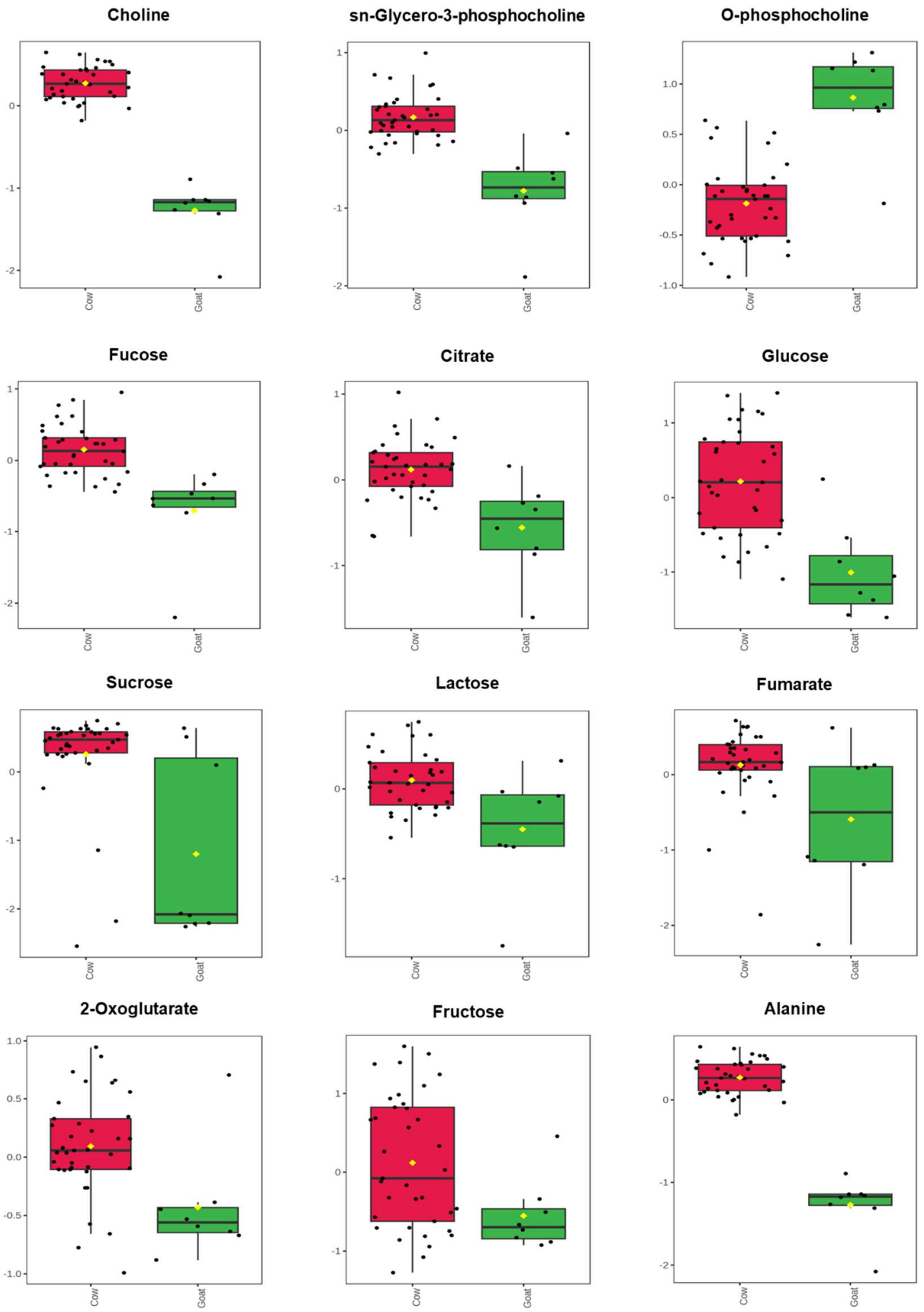

Figure S2: Box-whisker plots showing normalised levels of significantly differential metabolite in yoghurtsa mples from cow and goat milk. Boxes denote the interquartile range (IQR) between the bot-tom and top b oundaries (25th and 75th percentiles); the horizontal line inside the box denotes the median. The lower an d upper whiskers are the 5th and 95th percentiles, respectively;

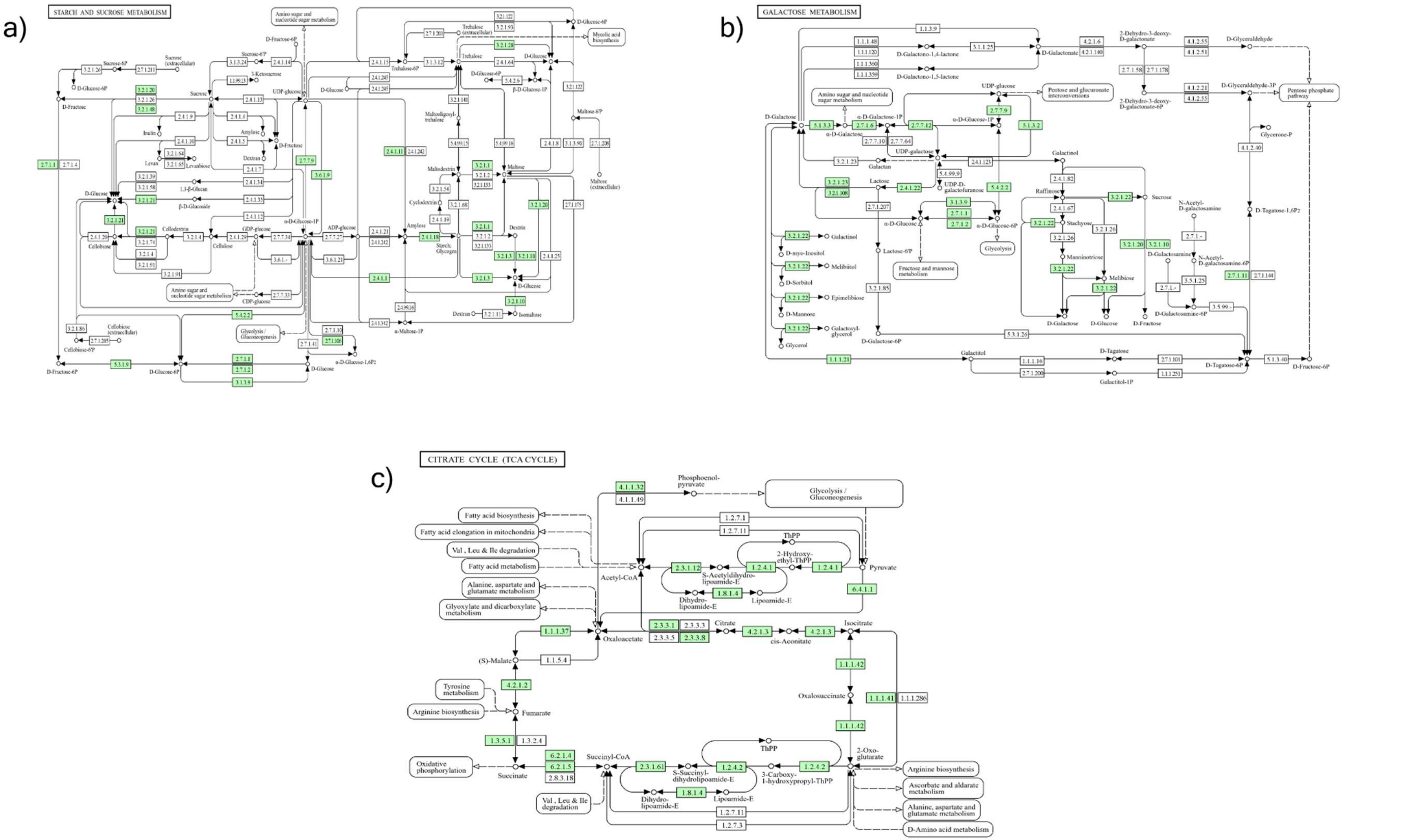

Figure S3: Snapshot of KEGG pathway maps for the a) starch and sucrose metabolism b) galactose metabolism and c) citrate cycle significantly altered in the sa mples of cow milk and goat milk yoghurt
